# Targeting NEDDylation is a Novel Strategy to Attenuate Cisplatin-induced Nephrotoxicity

**DOI:** 10.1158/2767-9764.CRC-22-0340

**Published:** 2023-02-13

**Authors:** Trace M. Jones, Claudia M. Espitia, Juan Chipollini, Benjamin R. Lee, Jason A. Wertheim, Jennifer S. Carew, Steffan T. Nawrocki

**Affiliations:** 1 Division of Hematology and Oncology, Department of Medicine, University of Arizona Cancer Center, Tucson, Arizona.; 2 Department of Urology, University of Arizona, Tucson, Arizona.; 3 Departments of Surgery and Biomedical Engineering, University of Arizona, Tucson, Arizona.

## Abstract

Although cisplatin remains a backbone of standard-of-care chemotherapy regimens for a variety of malignancies, its use is often associated with severe dose-limiting toxicities (DLT). Notably, 30%–40% of patients treated with cisplatin-based regimens are forced to discontinue treatment after experiencing nephrotoxicity as a DLT. New approaches that simultaneously prevent renal toxicity while improving therapeutic response have the potential to make a major clinical impact for patients with multiple forms of cancer. Here, we report that pevonedistat (MLN4924), a first-in-class NEDDylation inhibitor, alleviates nephrotoxicity and synergistically enhances the efficacy of cisplatin in head and neck squamous cell carcinoma (HNSCC) models. We demonstrate that pevonedistat protects normal kidney cells from injury while enhancing the anticancer activity of cisplatin through a thioredoxin-interacting protein (TXNIP)-mediated mechanism. Cotreatment with pevonedistat and cisplatin yielded dramatic HNSCC tumor regression and long-term animal survival in 100% of treated mice. Importantly, the combination decreased nephrotoxicity induced by cisplatin monotherapy as evidenced by the blockade of kidney injury molecule-1 (KIM-1) and TXNIP expression, a reduction in collapsed glomeruli and necrotic cast formation, and inhibition of cisplatin-mediated animal weight loss. Inhibition of NEDDylation represents a novel strategy to prevent cisplatin-induced nephrotoxicity while simultaneously enhancing its anticancer activity through a redox-mediated mechanism.

**Significance:**

Cisplatin therapy is associated with significant nephrotoxicity, which limits its clinical use. Here we demonstrate that NEDDylation inhibition with pevonedistat is a novel approach to selectively prevent cisplatin-induced oxidative damage to the kidneys while simultaneously enhancing its anticancer efficacy. Clinical evaluation of the combination of pevonedistat and cisplatin is warranted.

## Introduction

The alkylating agent cisplatin has been used for more than 40 years for the treatment of many tumor types including head and neck squamous cell carcinomas (HNSCC; ref. 1). Despite its prevalent use, severe dose-limiting toxicities (DLT) force a significant proportion of patients to discontinue cisplatin treatment (2). Nephrotoxicity is one of the most common DLTs associated with cisplatin therapy. This occurs due to the accumulation of the drug during glomerular filtration and tubular secretion, which results in higher concentration in the renal cortex than other organs (3). Enhanced hydration to decrease cisplatin accumulation in the kidney is the primary approach currently used to reduce nephrotoxicity. However, increased hydration is frequently insufficient to prevent renal toxicity (4). Prior studies have demonstrated that accumulation of cisplatin in the kidneys leads to oxidative stress–mediated tissue damage (5–7). Resultant acute renal failure with concomitant decreased renal blood flow or ischemia can compound the harmfulness. On the basis of this, various antioxidant compounds have been used to reduce kidney toxicity following cisplatin therapy. However, this toxicity reduction strategy may also reduce the antitumor activity of cisplatin as drug-induced reactive oxygen species (ROS) generation has been shown to play an important role in the ability of cisplatin to trigger apoptosis in malignant cells (3). New therapeutic approaches that maintain or preferably enhance cisplatin's anticancer efficacy while protecting the kidneys are desperately needed.

While the HNSCC staging, location, and genetic background of the tumor play a decisive role in the precise chemotherapy regimen, nearly all patients will receive cisplatin in some capacity (8). Although cisplatin has been proven to prolong median progression-free survival, its anticancer effects are often temporary and more than 50% of patients will experience a relapse within 2 years (9). Nephrotoxicity and acute kidney injury is seen, to some degree, in one-third of patients and increases risk of complication-related death by 10- to 15-fold (10). Compounding this issue is the reality that human papillomavirus (HPV)-negative (HPV−) HNSCC is strongly associated with the chronic abuse of alcohol and tobacco. Because of this, patients presenting with HPV− disease often have a multitude of comorbidities, making them less capable of tolerating toxicities associated with treatment. Thus, there exists a critical need to both improve the efficacy of platinum-based therapies and develop effective toxicity-mitigating strategies.

Posttranslational modification with the small ubiquitin-like molecule NEDD8 controls the activity of the cullin RING family of E3 ubiquitin ligases (CRL). The CRL family plays a critical role in the regulation of multiple cellular pathways including the oxidative stress response. The NEDDylation pathway is often highly overactivated in HNSCC tumors (11–14). We have previously shown that inhibiting NEDDylation greatly increases HNSCC sensitivity to cisplatin through the impairment of Cullin 4A (15). Accordingly, our study showed that the first-in-class NEDDylation inhibitor pevonedistat synergizes with cisplatin in both *in vitro* and *in vivo* models of HNSCC. Although pevonedistat is being clinically developed as an anticancer agent, recent studies have suggested that it may have additional applications for the protection of normal tissue from oxidative damage in models of polycystic liver disease and myocardial ischemia (16–18). Here we demonstrate for the first time that pevonedistat activates antioxidant defense mechanisms selectively in normal kidney cells, which alleviates cisplatin-induced nephrotoxicity while simultaneously improving the anticancer activity of cisplatin against HNSCC cells. Mechanistically, we show that this is driven by the differential expression of the pro-oxidative stress protein, thioredoxin-interacting protein (TXNIP). Importantly, the combination of pevonedistat and cisplatin significantly reduced kidney toxicity induced by cisplatin monotherapy and yielded long-term survival in 100% of HNSCC tumor-bearing mice. Taken together, these findings demonstrate that pevonedistat is a powerful chemosensitizer in HNSCC that simultaneously provides significant protection from cisplatin-induced oxidative stress in normal kidney tissue.

## Materials and Methods

### Cell Lines and Cell Culture

Renal proximal tubule epithelial cells (RPTEC) and HPV− HNSCC cell lines, FaDu and A253, were obtained from ATCC. Cell lines were authenticated using short tandem repeat DNA profiling. All experiments were conducted with *Mycoplasma*-free cells that had undergone less than five passages. RPTECs were cultured in renal epithelial cell basal medium (#PCS-400-030) supplemented with renal epithelial cell growth kit (#PCS-400-040) and 2% FBS (ATCC). Cancer cell lines were cultured in RPMI1640 medium supplemented with 10% FBS. All cells were grown at 37°C in 5% carbon dioxide. Harvesting of cells was performed by washing with PBS then incubating cells in a 0.25% trypsin, 2.21 mmol/L Ethylenediaminetetraacetic acid (EDTA), and 1X sodium bicarbonate solution at 37°C. Cell counts were performed using a Beckman Coulter Vi-CELL XR Cell Viability Analyzer (Beckman-Coulter).

### Chemicals and Reagents

Reagents were obtained from the following sources: Pevonedistat and cisplatin were purchased from SelleckChem. The antibodies anti-NEDD8 (ab81264), anti-KIM-1 (ab47635), anti-TXNIP (ab188865) anti-OCT2/SLC22A2 (ab170871), and anti-NRF2 (ab62352) were purchased from Abcam. The antibodies anti-CUL3 (#2759) and anti-β-Actin (#3700) were purchased from Cell Signaling Technology. Anti-CTR1/SLC31A1 (27499-1-AP) was obtained from Proteintech. Anti-β-Tubulin (#MA5-16308) and Prolong Gold antifade with DAPI were purchased from Thermo Fisher Scientific. Goat Anti-Rabbit horseradish peroxidase (HRP)-tagged secondary antibody (#111-035-144) was purchased from Jackson ImmunoResearch Laboratories.

### RNA Sequencing

RPTECs were subjected to 5 μmol/L pevonedistat for 48 hours. Pellets were collected, RNA was isolated, and subsequently subjected to RNA sequencing (RNA-seq) as described previously (19). qRT-PCR was used to independently validate RNA-seq expression results. A *P* value of 0.0001 was used in conjunction with a FDR cutoff of 0.0003 to determine significance. Differentially expressed genes following pevonedistat treatment are presented in Supplementary Table S1. All genes that changed at least 2-fold are included. Partek software was used to perform gene ontology pathway analysis. Experiments were performed in triplicate.

### Cell Viability Assays

3-(4,5-dimethylthiazol-2-yl)-2,5-diphenyltetrazolium bromide (MTT, #M5655, Sigma) viability assays were performed using at least three replicates. Puromycin was not present during any drug treatments. Cells were free of antibiotic selection for 1 week prior to experimentation. Cells were subjected to the indicated concentrations of drug for 72 hours. A total of 50 μL of 4 mg/mL MTT reagent was added to each well and plates were incubated at 37°C for 2 hours. Formazan precipitate was dissolved in 200 μL of DMSO and colorimetric readings were taken at 570 nm using a SpectraMax plate reader (Molecular Devices). The combination indices (CI) for pevonedistat and cisplatin were calculated using 72-hour MTT assay data and CompuSyn software (Combosyn, Inc.).

### Immunoblotting

Cells were treated for 24 or 48 hours with the indicated drug concentrations. Cells were harvested and lysed on ice for 1 hour in Triton X-100 lysis buffer (1% triton X-100, 150 nmol/L NaCl, 25 mmol/L Tris, pH 7.5) supplemented with protease and phosphatase inhibitors. Proteins were separated utilizing SDS-PAGE and transferred to a nitrocellulose membrane for blotting. Membranes were blocked in 5% BSA in Tris-buffered saline and Tween 20 (TBST) for 1 hour at room temperature. Membranes were incubated with primary antibodies overnight with agitation at 4°C. Membranes were then incubated with fluorescent secondary antibodies for 1 hour at room temperature. Imaging was performed using a fluorescent imager (LI-COR). β-actin and β-tubulin were used as loading controls. Quantification of protein bands was performed using ImageStudio densitometry software. Differences in band intensities for different immunoblots are due to varying exposure times.

### Immunocytochemistry

Cells were plated in 2-well chamber slides and treated with appropriate concentrations of drug for 48 hours. Cells were washed with PBS, fixed in 4% formaldehyde for 15 minutes, and permeabilized with 0.2% Triton X-100 for 10 minutes. Cells were incubated in blocking solution (5% horse serum, 1% goat serum in PBS) for 20 minutes. Anti-NRF2 primary antibody was diluted in blocking solution. Slides were incubated with primary antibody solution overnight at 4°C. Goat anti-rabbit AlexaFluro-544 secondary antibody was used to fluorescently visualize NRF2. DAPI was used to counterstain nuclei. Images were captured on a Zeiss Axio Vert.A1 microscope. ImageJ software was used to quantify signal intensity. Colocalization score was determined by measuring NRF2 mean signal intensity in regions that costained with DAPI.

### Short Hairpin RNA Knockdown of *TXNIP* and *NFE2L2 (NRF2)*

RPTECs were infected with lentiviral particles containing nontargeted scramble (vector) or target-specific short hairpin RNA (shRNA) directed at *TXNIP* (sc-270490-V) or *NFE2L2* (sc-37030-V) according to the manufacturer's protocol (Santa Cruz Biotechnology). Puromycin was used to select for successful transfection. Immunoblotting was used to assess knockdown efficiency. Transfected cells were subjected to indicated assays.

### CellROX Green Assay

Cells were plated in 2-well chamber slides. Cells were treated with appropriate drug concentrations for 48 hours. CellROX Green (Thermo Fisher Scientific) was added to a final concentration of 5 μmol/L. Cells were incubated in CellROX Green for 1 hour at 37°C. Cells were then washed with PBS and fixed in 3.7% formaldehyde for 15 minutes at room temperature. DAPI was used as a nuclear counterstain. Images were collected on a Zeiss Axio Vert.A1 microscope. ImageJ software was used to quantify CellROX Green mean signal intensity.

### qRT-PCR

Cells were treated with the indicated concentration of pevonedistat for 24 hours. Cells were collected and RNA was isolated using RNeasy mini kit (#74134, Qiagen) according to the manufacturer's instructions. cDNA was produced using SuperScript VILO cDNA synthesis kit (Invitrogen). Taqman primers for *GCLM*, *GCLC*, GSR*, TXNRD1*, *HMOX1*, *TXNIP, GAPDH*, *OCT2*, *CTR1*, and *β-Actin* were obtained from Thermo Fisher Scientific. Experiments were performed in triplicate.

### *In Vivo* Investigation

All animal studies were performed with the approval of the Institutional Animal Care and Use Committee of the University of Arizona (Tucson, AZ; 16-094) and were conducted in accordance with established guidelines. FaDu cells were collected, washed in PBS, and suspended in a 1:1 mixture of Hank's Balanced Salt Solution and Matrigel (#354234, Corning). Cells were implanted subcutaneously into the flanks of female nude (nu/nu) mice. Tumors were allowed to grow to a volume of 150 mm^3^. Mice were then assigned to treatment groups (*N* = 20 per group), and received vehicle control, pevonedistat 60 mg/kg s.c. five times a week (days 1–5, 8–12, 15–19, 22–26), cisplatin 3 mg/kg i.p. twice a week (days 1, 4, 8, 11, 15, 18, 22, 25), or the combination of both for four cycles of therapy. Pevonedistat and cisplatin were given simultaneously in the combination group on the same schedule as the monotherapies. Following treatment, 5 mice were sacrificed from each group. Tumors, kidneys, and serum were collected from these animals. Hematoxylin and eosin (H&E) staining was performed on each of these tissues. In addition, tissue was formalin fixed and paraffin embedded for IHC analyses. The remaining 15 mice in each group were monitored twice a week for an additional 72 days to establish long-term survival associated with the aforementioned therapies. GraphPad Prism software was used to generate a Kaplan–Meier survival curve and calculate median survival times.

### IHC

Paraffin-embedded tumor and kidney sections were deparaffinized in xylene, incubated in graded concentrations of ethanol, and rehydrated in a pH 7.5 PBS solution. Antigen retrieval was performed by microwaving the tissue sections for 5 minutes in a pH 8.0 EDTA solution. Endogenous peroxide blocking was performed by incubating tissue in a 3% hydrogen peroxide in methanol solution for 10 minutes. Protein blocking was performed by incubating samples in blocking solution (5% horse serum, 1% goat serum in PBS) for 20 minutes. Anti-NRF2, anti-TXNIP, anticleaved caspase-3, and anti-KIM-1 primary antibodies were diluted in blocking solution. Samples were incubated in primary antibody solutions overnight at 4°C. Slides were washed with PBS and incubated in an HRP-tagged secondary antibody for 90 minutes. Slides were then incubated with 3,3′-diaminobenzidine (#K3468, Dako) for 30 minutes. Samples were washed with water. Gill's hematoxylin (#GHS132, Sigma) was used to counterstain nuclei. Slides were imaged on a Zeiss Axio Vert.A1 microscope. ImageJ software with a Fiji plugin was used to analyze images.

### Renal Function Assessment

Blood urea nitrogen (BUN) and creatinine concentrations were determined using serum collected from treated mice utilizing a BUN colorimetric kit (Thermo Fisher Scientific) and creatinine kit (Crystal Chem), respectively.

### Measurement of *NRF2* Activity

*NRF2* activity was determined by using the X-MAN *NFE2L2* (*NRF2*) NanoLuc reporter kit of HCT116 cells (Horizon Discovery). Cells were plated in 96-well plates and treated with increasing concentrations of pevonedistat for 12 hours. Following treatment, Nano-Glo luciferase reagent was added to each well and luminescence was measured on a Molecular Devices plate reader.

### Statistical Analyses

Statistical significance of results was determined by performing the Student *t* test, one-way ANOVA, two-way ANOVA, or Kaplan–Meier analysis where appropriate. Differences were considered significant at *P* < 0.05. Prism GraphPad was used to perform all statistical tests.

### Data Availability

The data generated in this study are available within the article and its Supplementary Data. RNA-seq files are available through NCBI Sequence Read Archive database BioProject accession number PRJNA924768.

## Results

### Inhibition of NEDDylation is Therapeutically Selective

**FIGURE 1:**
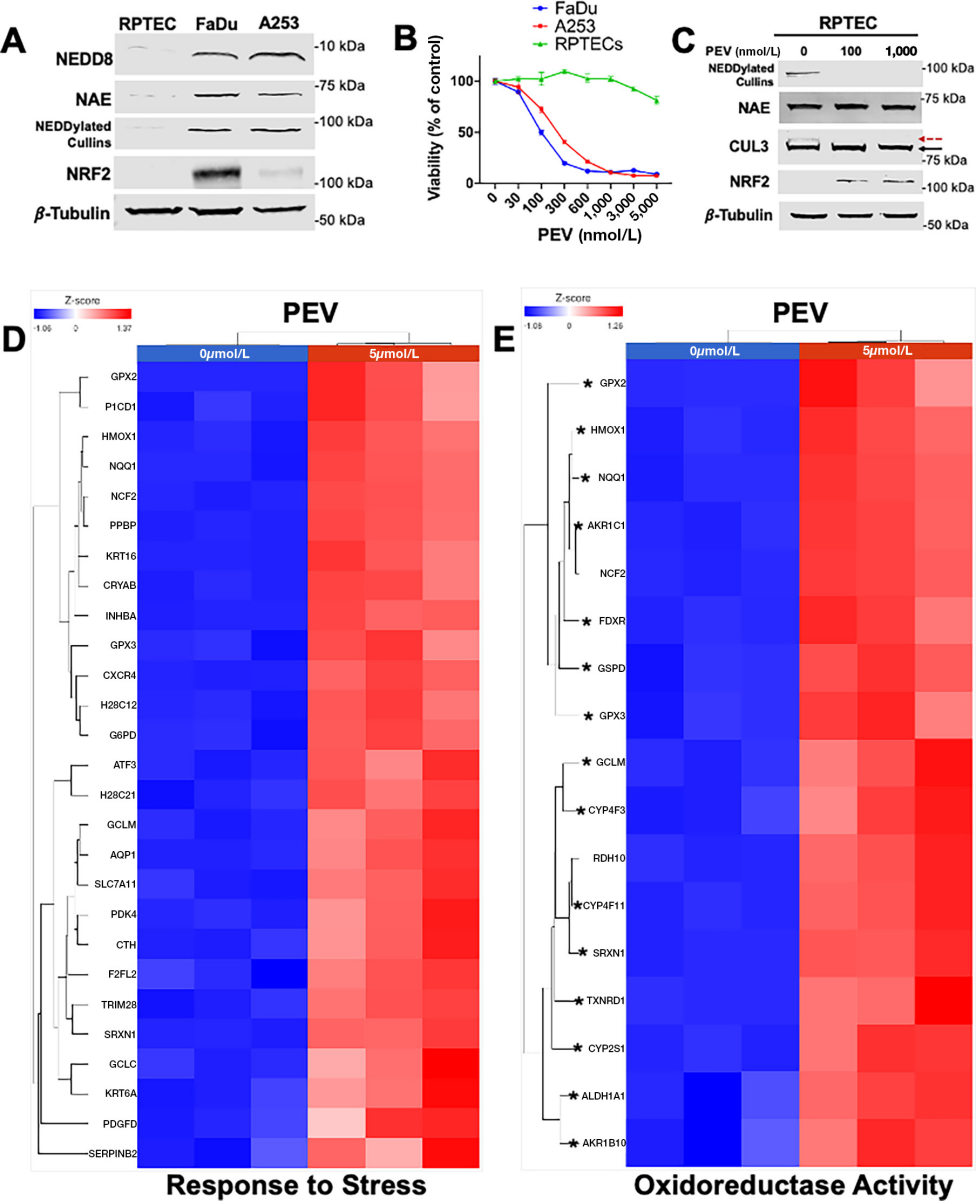
Pevonedistat activates protective oxidative stress response pathways in normal RPTECs. **A,** Proteins associated with NEDDylation are overexpressed in HNSCC cells. RPTEC, FaDu, and A253 cells were subjected to immunoblotting to determine the basal levels of NEDD8, NAE, NEDDylated cullins, and NRF2. β-Tubulin was used as a loading control. **B,** Pevonedistat does not decrease normal kidney cell viability. RPTEC, FaDu, and A253 cells were treated with the indicated concentrations of pevonedistat for 72 hours. MTT assays were used to determine cell viability. Mean ± SD, *n* = 4. **C,** Pevonedistat decreases cullin NEDDylation and stabilizes NRF2 in RPTEC. RPTECs were incubated with the indicated concentrations of pevonedistat for 72 hours. Immunoblotting was used to monitor changes in NEDDylated cullins, NAE, NEDDylated (red dotted arrow) and un-NEDDylated (black arrow) Cullin-3, and NRF2. β-Tubulin was used as a loading control. **D** and **E,** RPTECs were treated with 5 μmol/L pevonedistat for 48 hours. RNA-seq determined that the response to stress and oxidoreductase activity gene pathways were among the most highly enriched ontologies in RPTECs treated with pevonedistat.

To establish the basal expression status of NEDDylation-associated proteins, we performed immunoblotting using two HPV− HNSCC cell lines (FaDu and A253) and normal RPTECs (Fig. 1A). Free NEDD8, NEDD8-activating enzyme (NAE), and NEDD8-conjugated cullin levels were all found to be higher in HNSCC cell lines when compared with RPTECs. In addition, baseline levels of NRF2 were also shown to be significantly higher in HNSCC cells compared with RPTECs, confirming the dysregulation of NRF2 in HNSCC. We next assessed cellular viability following treatment with a variety of concentrations (0–5 μmol/L) of pevonedistat (Fig. 1B). Interestingly, RPTECs were dramatically more resistant to pevonedistat compared with the FaDu and A253 HNSCC cell lines. To further evaluate the protein-directed pharmacodynamics of pevonedistat, we performed immunoblotting with RPTECs treated with 100 and 1,000 nmol/L of pevonedistat (Fig. 1C). Loss of NEDDylated cullins was evident at both concentrations. Specifically, CUL3 displayed a complete loss of its NEDDylated form. In agreement with this result, NRF2 was shown to be stabilized in response to pevonedistat treatment. Finally, expression of NAE1, the functional target of pevonedistat, was not affected by the drug.

### Pevonedistat Activates Multiple Gene Pathways Associated with the Oxidative Stress Response in Normal Kidney Cells

To determine the genome-wide effects of pevonedistat treatment on normal kidney cells, we subjected RPTECs to pevonedistat for 48 hours and performed RNA-seq analysis (Supplementary Table S1). We also investigated gene networks that were significantly altered using gene ontology analyses. Two of the most highly enriched pathways in pevonedistat-treated RPTECs were the response to stress and oxidoreductase activity gene ontologies, indicating that pevonedistat is a significant regulator of the cellular stress response in normal kidney cells (Fig. 1D and E). To validate our RNA-seq results, we performed qRT-PCR on the key oxidative stress–related genes *GCLM*, *HMOX1*, and *TXNRD1* (Supplementary Fig. S1). qRT-PCR analysis revealed similar fold changes to those observed in our RNA-seq experiments. Further analysis revealed that a large portion of the upregulated genes within these gene ontologies are direct transcriptional targets of the oxidative stress response transcription factor, *NRF2* (Supplementary Table S2). These results reveal that pevonedistat treatment upregulates multiple genes that alleviate oxidative stress in normal kidney cells.

### Pevonedistat Treatment Significantly Increases NRF2 Activity

**FIGURE 2:**
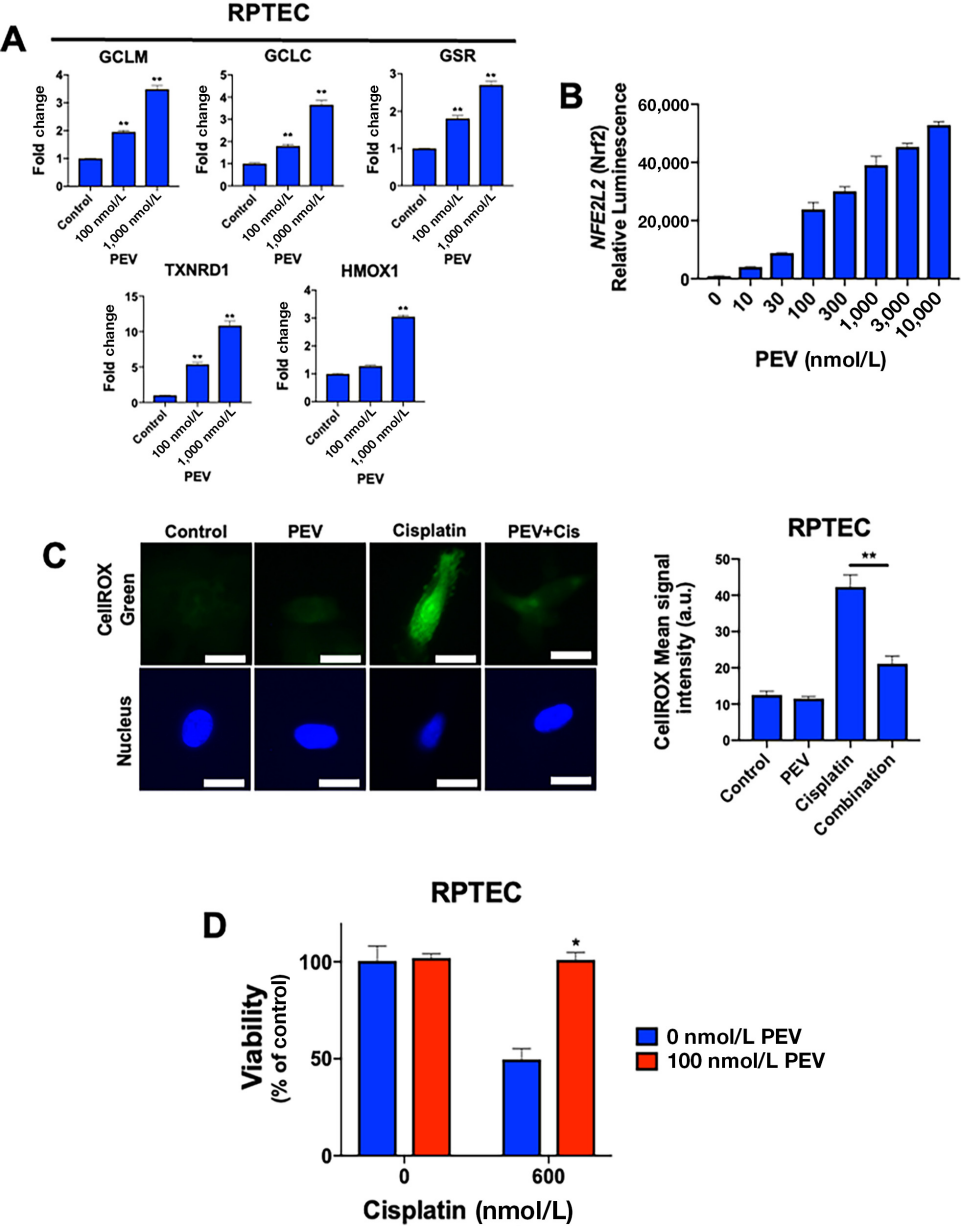
Pevonedistat provides protection from cisplatin in RPTECs. **A,** NRF2 target genes are significantly upregulated following pevonedistat treatment. RPTECs were incubated with the indicated concentrations of pevonedistat for 48 hours. qRT-PCR was used to determine changes in *GCLM*, *GCLC*, *GSR*, *TXNRD1*, and *HMOX1* gene expression. *GAPDH* was used as a house keeping gene for normalization. Mean ± SD, *n* = 3; **, *P* < 0.01; indicates significant difference from control condition. **B,** Pevonedistat-induced NRF2 is transcriptionally active. NRF2 NanoLuc reporter cells were treated with the indicated concentrations of pevonedistat for 12 hours. *NRF2* activity was measured using the X-MAN Horizon reporter kit. Luminescence was quantified using a Molecular Devices plate reader. Mean ± SD, *n* = 3. **C,** Pevonedistat reduces the levels of cisplatin-induced oxidative stress. RPTECs were treated with 150 nmol/L pevonedistat, 20 μmol/L cisplatin, or the combination for 48 hours. Cells were then stained with CellROX Green and imaged. Mean signal intensity was quantified using ImageJ. DAPI was used as a nuclear counterstain. Mean ± SEM, *n* = 30; **, *P* < 0.01. **D,** Pevonedistat reduces RPTEC cell death in response to cisplatin treatment. RPTECs were incubated with the indicated concentrations of cisplatin and pevonedistat for 72 hours. Cell viability was determined using an MTT assay. Mean ± SD, *n* = 3; *, *P* < 0.05; indicates a significant difference from untreated cells.

We next sought to evaluate whether stabilized NRF2 was transcriptionally active following pevonedistat treatment. We first performed qRT-PCR to quantify drug-induced changes in the mRNA levels of classical *NRF2* target genes (Fig. 2A). Genes in the glutathione (*GCLM*, *GCLC*, *GSR*), thioredoxin (*TXNRD1*), and heme oxygenase (*HMOX1*) pathways were all shown to be significantly upregulated by pevonedistat in a dose-dependent manner. To further confirm the transcriptional activity of *NRF2*, we conducted immunocytochemistry analyses (Supplementary Fig. S2A). We determined that RPTECs exposed to pevonedistat, both as a single agent and in combination with cisplatin, displayed significantly elevated levels of NRF2 (Supplementary Fig. S2B). Importantly, these cells also displayed significant nuclear localization of NRF2 (Supplementary Fig. S2C). Notably, treatment with cisplatin alone did not elevate expression or localization of NRF2 to the nucleus. Finally, using nano-luc reporter *NRF2* transfected cells, we determined that treatment with pevonedistat resulted in significantly increased *NRF2* transcriptional activity (Fig. 2B). To determine whether NRF2 is required for pevonedistat-mediated protection of RPTECs form cisplatin, we silenced its expression using lentiviral shRNA (Supplementary Fig. S3A). Knockdown of NRF2 significantly ablated the ability of pevonedistat to reduce cisplatin toxicity in RPTECs (Supplementary Fig. S3B). Taken together, these experiments demonstrate that pevonedistat significantly stabilizes NRF2 and activates its associated antioxidant response pathways and that NRF2 expression significantly contributes to the protective effects of pevoendistat against cisplatin toxicity.

### Cisplatin-induced Oxidative Stress is Significantly Diminished by Pevonedistat in RPTECs

After establishing that pevonedistat activates the *NRF2* transcriptional network, we sought to determine whether drug treatment was sufficient to abrogate cisplatin-induced oxidative stress in RPTECs. We utilized CellROX Green, a cell-permeant dye that fluoresces after it has been oxidized by ROS, to quantify ROS levels (Fig. 2C). Treatment with single-agent cisplatin resulted in high levels of CellROX fluorescence. Cotreatment of RPTECs with pevonedistat and cisplatin significantly reduced this signal. To determine whether this reduction in oxidative stress resulted in improved primary cell survival, we performed MTT cellular viability assays. RPTECs cotreated with pevonedistat and cisplatin showed protection from cisplatin-induced cell death when compared with cells treated with cisplatin alone (Fig. 2D). Finally, we conducted formal CI analysis using CompuSyn software to determine whether combination drug activity was truly antagonistic in primary kidney cells (Supplementary Table S3). CI analyses demonstrated that the interaction of pevonedistat and cisplatin is highly antagonistic across multiple concentrations in RPTECs. Membrane transporters such as organic cation transporter-2 (OCT2) and copper transporter-1 (CTR1) mediate the cellular transport of cisplatin (20). OCT2 is specifically expressed in the kidneys and has been demonstrated to be a potential actionable target to prevent toxicity induced by platinum chemotherapy (21, 22). To investigate whether pevonedistat modulates the expression of these transporters, we conducted qRT-PCR and immunoblotting to evaluate their expression. We did not observe significant drug-induced modulation in the expression of either OCT2 or CTR1. This suggests that pevonedistat likely does not protect against cisplatin-mediated nephrotoxicity through a direct effect on transporter levels (Supplementary Fig. S4A and S4B). Collectively, these data demonstrate that pevonedistat promotes an antioxidant response in normal kidney cells resulting in protection from cisplatin-induced oxidative stress and toxicity.

### TXNIP is Differentially Regulated in Primary Kidney Cells and Cancer Cells Following Pevonedistat Treatment

**FIGURE 3:**
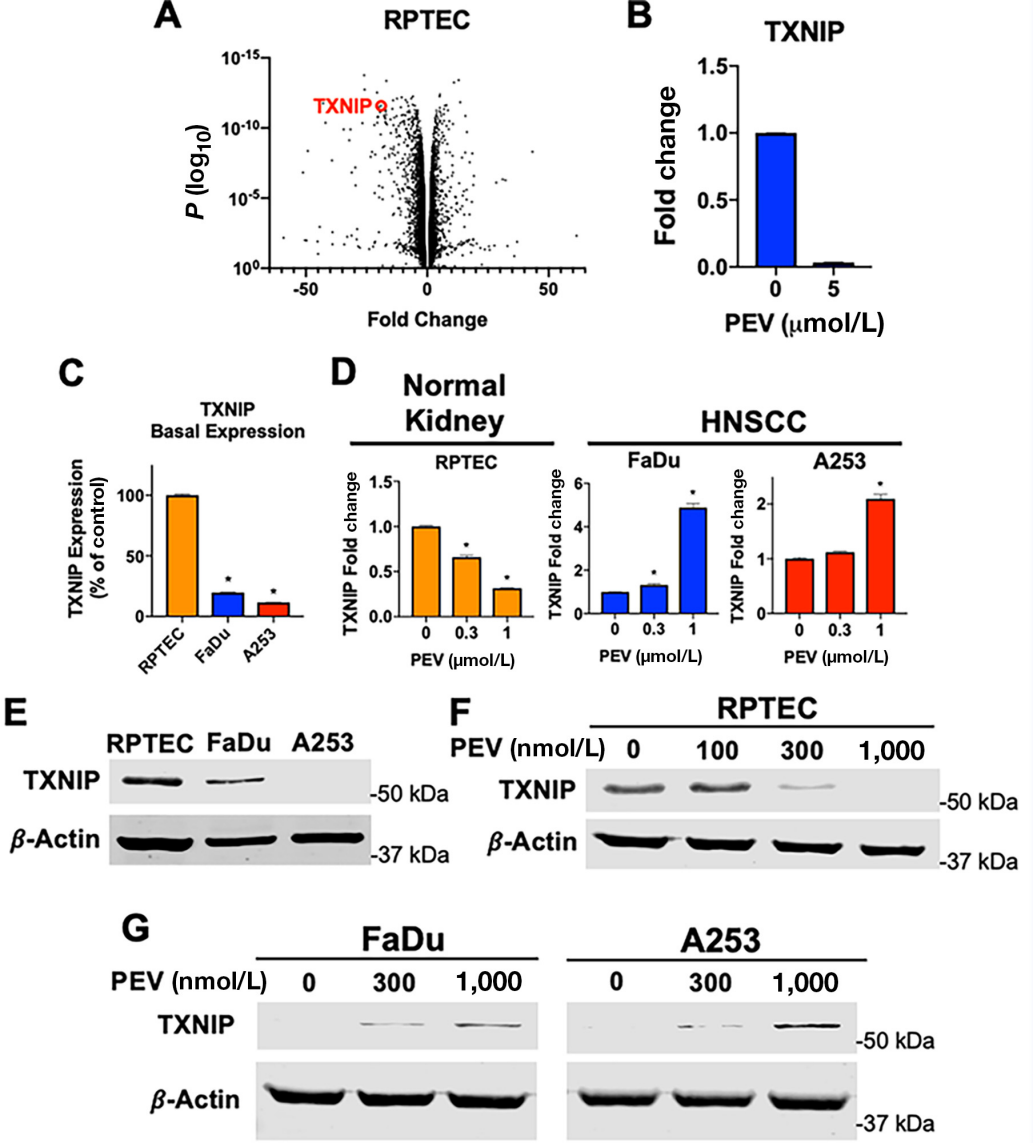
TXNIP is differentially regulated in HNSCC and normal kidney cells in response to pevonedistat treatment. **A,***TXNIP* is among the most significantly downregulated genes in RPTECs treated with pevonedistat. Genes exhibiting fold changes greater than ±3.5 were plotted on a volcano plot. *TXNIP* displayed a fold change of −19.05 with a *P* value of 2.65E-12. **B,** RNA samples used for RNA-seq were subjected to qRT-PCR to confirm *TXNIP* downregulation. **C,***TXNIP* is significantly lower in HNSCC cells compared with RPTECs. RNA was extracted from RPTEC, FaDu, and A253 cells. qRT-PCR was performed to determine basal *TXNIP* expression. Mean ± SD, *n* = 3; *, *P* < 0.05; indicates significant difference from RPTEC expression. **D,***TXNIP* is differentially regulated following pevonedistat treatment. RPTEC, FaDu, and A253 cells were treated with the indicated concentrations of pevonedistat. RNA was extracted and qRT-PCR was performed to determine *TXNIP* expression. *GAPDH* was used as a housekeeping gene. Mean ± SD, *n* = 3; *, *P* < 0.05. **E,** TXNIP protein levels are lower in HNSCC cells. Immunoblotting was performed to determine the basal protein levels of TXNIP. **F** and **G,** RPTEC, FaDu, and A253 cells were treated with indicated concentrations of pevonedistat for 48 hours. Immunoblotting was used to determine changes in TXNIP protein levels. β-Actin was used as a loading control.

While much focus has been placed on upregulating stress response genes in normal kidney cells to mitigate oxidative damage induced by cisplatin therapy, very little has been done to investigate pro-oxidative stress and proapoptotic genes which may also play a significant role. Interestingly, our RNA-seq data determined that TXNIP, a prominent oxidative stress inducer, was among the most highly significantly downregulated genes in response to pevonedistat treatment in RPTECs (Fig. 3A). We confirmed this finding by qRT-PCR analysis (Fig. 3B). Notably, TXNIP acts as a tumor suppressor and is often downregulated in cancer cells (23). TXNIP's primary function in normal cells is to maintain oxidative homeostasis by binding to reduced thioredoxin, disallowing thioredoxin's ROS quenching capabilities (24). Furthermore, TXNIP expression is repressed by NRF2 (25). We investigated TXNIP levels in RPTEC and HNSCC cells by qRT-PCR (Fig. 3C). TXNIP levels were found to be significantly lower in HNSCC cells compared with RPTEC. We next sought to determine how TXNIP expression changes in response to pevonedistat therapy in RPTEC and HNSCC cells. We performed qRT-PCR on RPTECs, FaDu, and A253 cells after treatment with pevonedistat (Fig. 3D). RPTECs demonstrated a dose-dependent decrease of TXNIP expression, which was consistent with our RNA-seq data. Interestingly, TXNIP levels in the malignant FaDu and A253 cells were significantly upregulated in response to pevonedistat treatment. We next conducted immunoblotting experiments to confirm that these pharmacodynamic changes also occurred at the protein level. Basal levels of TXNIP protein were found to be significantly higher in RPTECs compared with FaDu or A253 HNSCC cell lines (Fig. 3E). As expected, treatment with pevonedistat resulted in a dose-dependent reduction of TXNIP protein expression in RPTECs (Fig. 3F) and increased TXNIP protein expression in both HNSCC cell lines (Fig. 3G). Altogether, these findings indicate that TXNIP is differentially regulated in RPTECs and HNSCC cells.

### TXNIP Regulates the Survival of Kidney Cells Following Cisplatin Treatment

**FIGURE 4:**
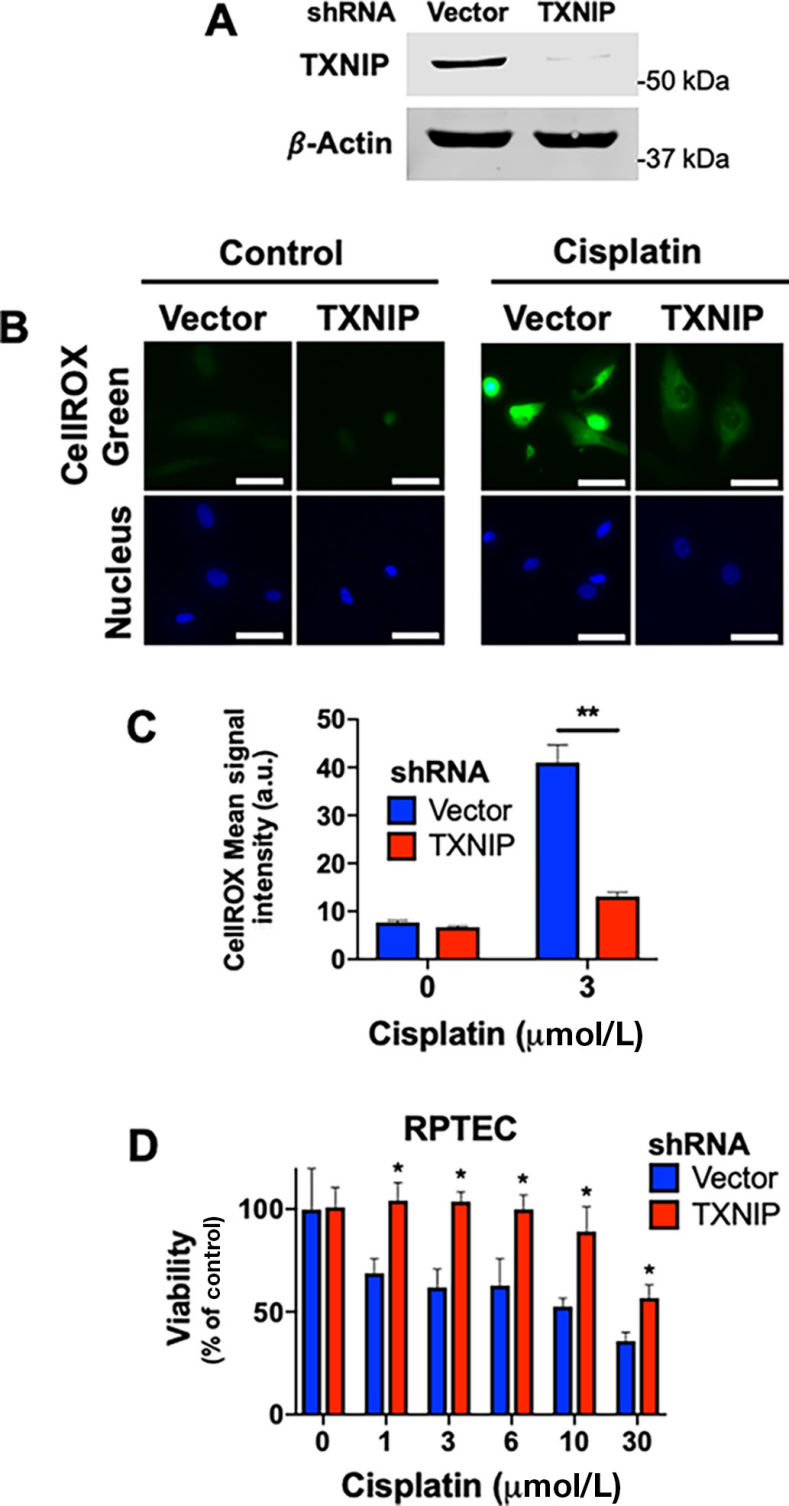
Loss of TXNIP protects RPTECs from cisplatin-induced ROS and toxicity. **A,** RPTECs were transfected with lentiviral vector control or TXNIP shRNA particles. Puromycin was used to select for successful transfection. Immunoblotting was used to determine knockdown efficiency. β-Actin was used as a loading control. **B,** shRNA TXNIP and vector control RPTECs were treated with the indicated concentrations of cisplatin for 48 hours. Cells were stained with CellROX Green and imaged. **C,** ImageJ was used to determine the average CellROX Green signal intensity. Mean ± SEM, *n* = 45 cells; *, *P* < 0.05. **D,** shRNA TXNIP and vector control RPTECs were subjected to 72-hour treatment with the indicated cisplatin concentrations. MTT assay was used to determine cell viability. Mean ± SD, *n* = 3; *, *P* < 0.05.

To further investigate the role of TXNIP levels as a driver of cisplatin-induced renal cell toxicity, we used lentiviral shRNA to knockdown its expression in RPTECs. Knockdown efficiency was determined by immunoblotting (Fig. 4A). Vector control and TXNIP knockdown cells were treated with 3 μmol/L cisplatin treatment for 48 hours and ROS levels were quantified by CellROX Green staining (Fig. 4B and C). These assays showed that TXNIP knockdown cells displayed significantly lower levels of ROS than their vector control counterparts. We then subjected these cells to 72-hour treatment with a range of cisplatin concentrations (0–30 μmol/L) and quantified cell viability by MTT assay (Fig. 4D). TXNIP shRNA cells displayed significantly higher levels of cell viability across all concentrations of cisplatin, demonstrating that TXNIP plays an important role in regulating cisplatin-induced kidney cell toxicity. Together, these data provide evidence that the downregulation of TXNIP by pevonedistat in RPTEC is a major contributor to the observed reduction in cisplatin-induced cytotoxicity.

### Pevonedistat Reduces Cisplatin-induced Toxicity *In Vivo*

**FIGURE 5:**
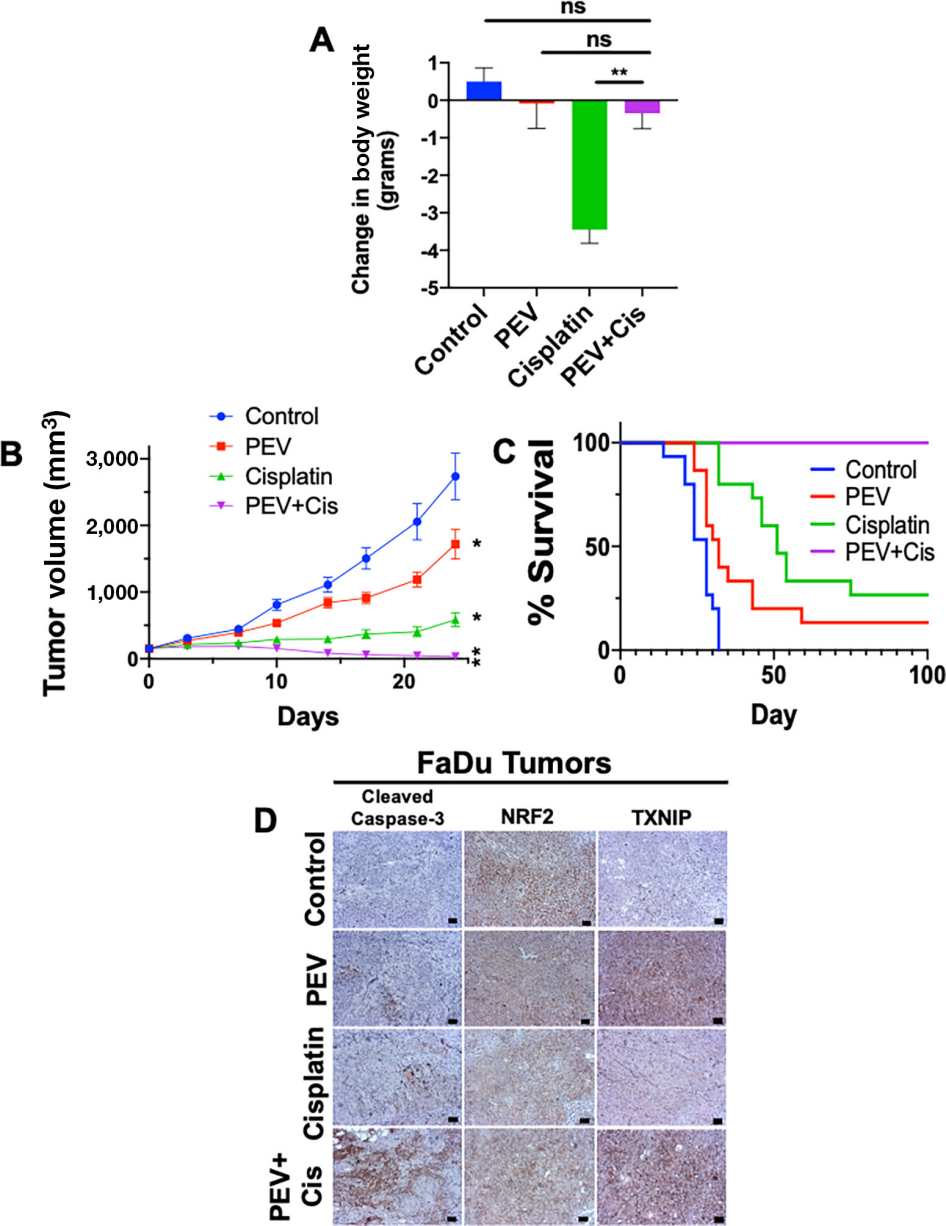
Pevonedistat increases the *in vivo* anticancer activity of cisplatin while mitigating cisplatin-mediated nephrotoxicity. **A,** FaDu tumors were established in nude mice. When tumors reached approximately 150 mm^3^ in size, mice were treated with vehicle control, 60 mg/kg pevonedistat s.c. 5 consecutive days per week, 3 mg/kg cisplatin i.p. twice a week, or the combination for 4 weeks. Mice receiving pevonedistat and cisplatin lost significantly less body weight than mice treated with cisplatin alone. Change in body weight was calculated by subtracting starting mouse weight and tumor weight from the final weight of the mouse. Mean ± SEM, *n* = 15; **, *P* < 0.01. **B,** The combination of pevonedistat and cisplatin yields significant tumor regression. Mean ± SEM, *n* = 15; ** signifies a significant difference from single-agent therapies; * signifies a significant difference from vehicle control; *P* < 0.05. **C,** The combination of pevonedistat and cisplatin yields long-term survival in tumor-bearing mice. Mice were monitored until day 100 of the experiment. No signs of toxicity or tumors were observed in combination treated mice on day 100. Overall survival was determined by Kaplan–Meier survival analysis, *n* = 15 per group. **D,** Pevonedistat increases the proapoptotic effects of cisplatin by increasing TXNIP levels in HNSCC tumors. Excised tumors were immunohistochemically stained and imaged for cleaved caspase-3, NRF2, and TXNIP expression. Scale bar, 20 μm.

We next investigated the potential of pevonedistat to protect against toxicities associated with cisplatin therapy in mice while enhancing its anticancer effects. FaDu HNSCC cells were implanted into the flanks of nude mice. Mice were treated with pevonedistat (60 mg/kg s.c., five times per week), cisplatin (3 mg/kg i.p., twice a week), or the combination for 4 weeks. To monitor systemic toxicity, mice were observed and weighed twice a week during treatment. At the cessation of therapy, mice in the control and pevonedistat groups displayed similar weight, while mice treated with cisplatin monotherapy lost a significant amount of weight. Importantly, mice treated with the combination maintained their prestudy weight (Fig. 5A). These data suggests that pevonedistat acts as a mitigator of the toxicities associated with cisplatin treatment. Furthermore, mice receiving pevonedistat and cisplatin combination therapy experienced significant and sustained tumor regression (Fig. 5B). Mice were monitored for delayed toxicities and tumor regrowth until day 100 and mice in the combination group displayed 100% survival with no tumor regrowth and no observable toxicities (Fig. 5C). The combination of pevonedistat and cisplatin yielded a median survival time double that of single-agent cisplatin (Supplementary Table S4). Taken together, these results indicate that pevonedistat alleviates toxicities associated with cisplatin therapy while also improving its antitumor efficacy.

### Pharmacodynamic Analyses Demonstrate That Pevonedistat Protects Against Cisplatin-induced Kidney Damage

**FIGURE 6:**
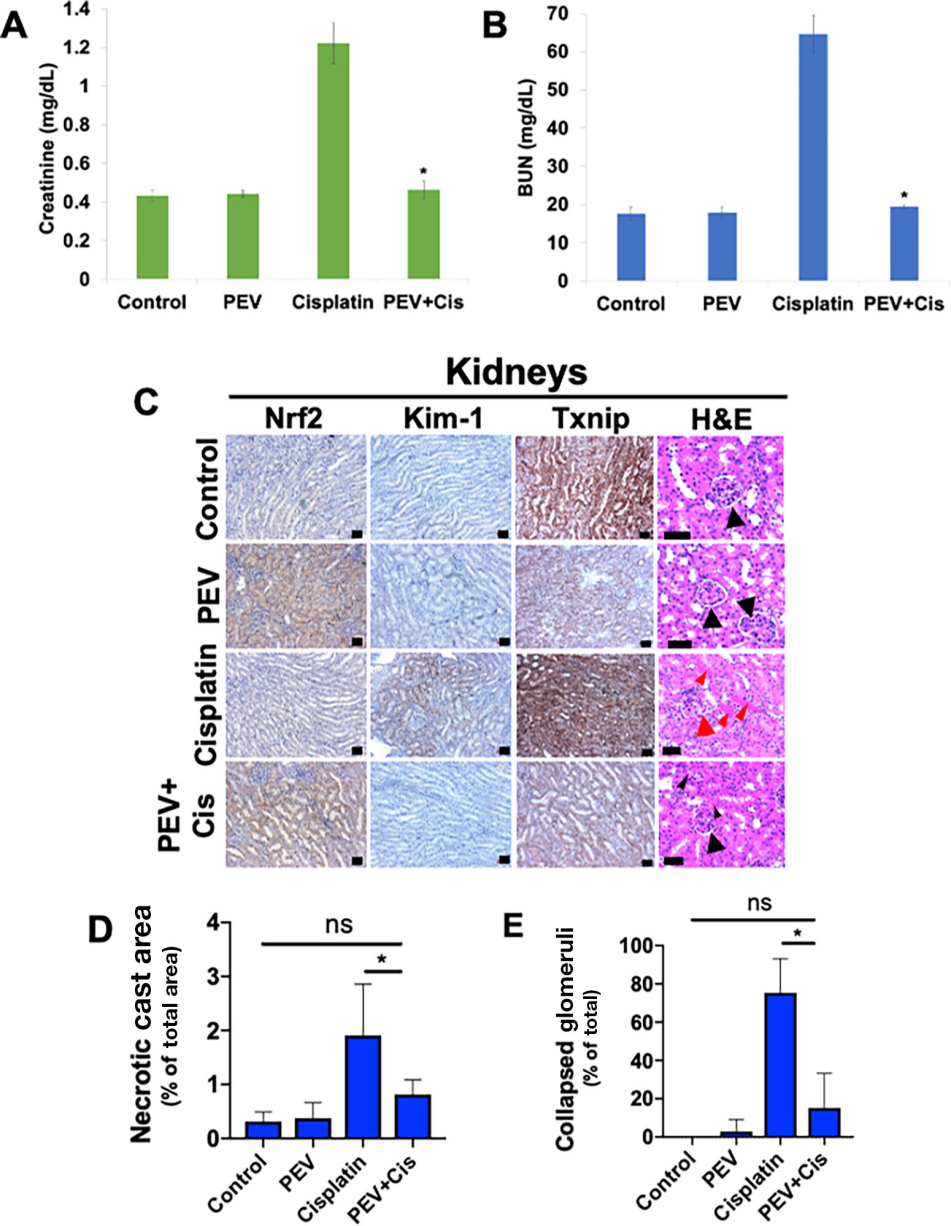
Pevonedistat protects kidneys from cisplatin-induced injury. **A** and **B,** Pevonedistat reduced the levels of cisplatin-induced creatinine and BUN. Serum creatinine and BUN were measured in mice treated with vehicle control, pevonedistat, cisplatin, or the combination. Mean ± SD, *n* = 3, * indicates a significant difference from cisplatin treated mice; *P* < 0.05. **C,** Pevonedistat mitigates kidney damage caused by cisplatin in mice. Kidneys were excised and paraffin embedded. Nrf2, Kim-1, and Txnip levels were measured by IHC. Physical signs of damage were evaluated and quantified via H&E staining. Large black arrows indicate intact glomeruli. Large red arrows represent collapsed glomeruli. Small red arrows indicate necrotic casts. Scale bar, 20 μm. **D,** Necrotic cast area was measured using ImageJ. Cast area was divided by total image area. Mean ± SD, *n* = 5 images per condition. *, *P* < 0.05. **E,** Collapsed glomeruli were counted in each image. The number of collapsed glomeruli was divided by the total number counted for each image. Mean ± SD, *n* = 5 images per condition. *, *P* < 0.05.

Tumors and kidneys were excised from mice in each treatment group for IHC evaluation of *in vivo* pharmacodynamics. Tumors that were treated with the combination of pevonedistat and cisplatin displayed significantly higher levels of cleaved caspase-3 compared with control and monotherapies (Fig. 5D; Supplementary Fig. S5). NRF2 expression remained relatively constant in tumor tissue following treatment as NRF2 exhibits high basal expression in FaDu cells. TXNIP levels were highly increased in tumor tissue treated with pevonedistat and combination therapy (Fig. 5D; Supplementary Fig. S3). To evaluate the effects of drug treatment on established markers of nephrotoxicity, we measured the levels of serum BUN and creatinine (Fig. 6A and B). Serum levels of BUN and creatinine were both increased following cisplatin treatment. However, pevonedistat was able to significantly block cisplatin-induced BUN and creatinine accumulation. Expression of Nrf2, Txnip, and the kidney injury marker kidney injury molecule-1 (Kim-1) were also determined in excised murine kidney cross-sections (Fig. 6C; Supplementary Fig. S5). These analyses showed that kidneys from mice receiving pevonedistat and combination therapies displayed significantly higher levels of Nrf2. Kim-1 expression was elevated in kidneys from mice receiving cisplatin monotherapy. However, the addition of pevonedistat blocked cisplatin-induced Kim-1 upregulation (Fig. 6C; Supplementary Fig. S5). In agreement with our *in vitro* findings, kidneys exposed to pevonedistat also displayed decreased levels of Txnip, (Fig. 6C; Supplementary Fig. S5). Finally, we stained kidneys from each treatment group with H&E to visualize physical evidence of kidney damage (Fig. 6C). Kidneys taken from mice in the control and pevonedistat groups displayed intact glomerular capsules with little to no cast formation. Kidneys exposed to single agent cisplatin showed glomerular capsules in various stages of decay and cast formation indicative of necrotic tissue (Fig. 6C). However, introduction of pevonedistat reduced cast formation (Fig. 6D) and glomerular degeneration (Fig. 6E). Taken together, these experiments demonstrate that pevonedistat is capable of mitigating damage to the kidney caused by cisplatin therapy, while simultaneously augmenting its antitumor efficacy.

## Discussion

Cisplatin remains a primary chemotherapeutic agent used in the clinical management of HPV− HNSCC and other malignancies. More than 30% of patients receiving high-dose cisplatin therapy experience an acute kidney injury, an adverse event that carries a 50% mortality rate (10). Given that the vast majority of patients with HPV− HNSCC have a history of chronic alcohol or tobacco abuse, patients often present with comorbidities which can be further exacerbated by the off-target effects of cisplatin. In addition, more than 65% of patients with HNSCC that receive cisplatin therapy develop metastatic or recurrent disease (26). Current treatment modalities yield a median overall survival of less than 1 year in this patient population (27, 28). Therefore, there is an urgent clinical need for strategies that can alleviate toxicity while also improving the anticancer activity of cisplatin in patients with HNSCC.

A recent study found that pevonedistat reduces acute cisplatin-induced nephrotoxicity through downregulation of inflammatory mediators such as IL6, TNFα, IL1β, and NFκB (29). They also concluded that the protection was not due to alterations in the expression of the antioxidant glutathione. While we agree in principle with this report in that pevonedistat provides renal protection from cisplatin injury, there are important distinctions between this earlier study and our current findings. First, the overall experimental designs are quite different. The prior study induced kidney injury using a single high dose of cisplatin (25 mg/kg) that is far above the dose levels that are administered to humans receiving cisplatin chemotherapy. In contrast, our study intentionally utilized a therapeutic dose of 3 mg/kg cisplatin given twice a week over 4 weeks to better model the nature and degree of cisplatin toxicity experienced by patients with cancer when treated with cisplatin-containing regimens. In addition, our mechanistic investigation determined for the first time that NRF2, oxidative stress, and TXNIP play a significant role in pevonedistat-mediated renal protection from cisplatin. The differences in the identified factors underlying the protective effect of pevonedistat in this context between these two studies are likely due to the distinct treatment approaches that were used: high-dose acute drug treatment in the earlier study versus chronic exposure to a clinically relevant therapeutic dose of cisplatin in our investigation. Given this, we feel that our current findings provide significant new insights into the mechanisms driving the ability of pevonedistat to protect the kidneys from the toxicity associated with chronic exposure to cisplatin that is experienced by patients undergoing cancer chemotherapy with this drug.

Consistent with our findings, previous studies have reported that the upregulation of antioxidant pathways in kidneys is effective in countering the toxic side effects of cisplatin (30, 31). However, simply activating antioxidant pathways indiscriminately and systemically has the potential to interfere with the antitumor activity of cisplatin. Our findings demonstrate that the first-in-class NEDDylation inhibitor, pevonedistat, mitigates the off-target, kidney-damaging effects of cisplatin while also greatly enhancing its antitumor activity. The cytoprotective effects of pevonedistat that we observed in normal renal cells are consistent with reports from other groups that showed that inhibition of NEDDylation protects other types of normal cells against various types of oxidative injuries. Disrupting NEDDylation reduced lipid peroxidation, ROS production, and apoptosis in liver cells in models of nonalcoholic fatty liver disease (18, 32, 33). Another investigation demonstrated that pevonedistat has a strong cardioprotective effect against myocardial ischemia-reperfusion injury, a condition that is associated with oxidative stress–induced cell death of normal heart tissue (16). In a similar manner, pevonedistat also provided a neuroprotective effect in spinal cord neurons subjected to oxidative stress caused by spinal cord ischemia-reperfusion injury (17). The proposed mechanisms of protection in these tissues are different than what we discovered in our renal toxicity study. However, these collective findings strongly indicate that pevonedistat can protect normal tissue from ROS stress–induced injury and cell death.

Interestingly, we discovered that TXNIP is differentially regulated in normal kidney cells and HNSCC cells in response to pevonedistat treatment. TXNIP belongs to the α-arrestin family of proteins and its inappropriate regulation in cancer cells has been reported previously (34). TXNIP binds both reduced cytosolic and mitochondrial thioredoxin and this inhibits further ROS quenching by thioredoxin (24, 35). Because of this function, increased expression of TXNIP renders cells more vulnerable to oxidative insult and apoptosis. TXNIP is classified as a tumor suppressor and its basal expression is frequently lost in cancer (36, 37). Previous studies have shown that restoration of TXNIP expression sensitizes cancer cells to cytotoxic chemotherapeutics (38, 39). However, high expression of TXNIP has also been classified as a biomarker for oxidative stress and myocardial ischemia in heart tissue (40, 41). While multiple mechanisms of regulation of TXNIP exist, NRF2 has been shown to be a potent repressor of this gene in normal cells (25, 42). HNSCC is often characterized by the inappropriate expression and activity of NRF2, resulting in altered regulation of its transcriptional activation and repression of its targets (43–45). Thus, normal mechanisms of TXNIP regulation by NRF2 are likely lost in HNSCC cells.

A limitation of our study is that we did not measure cisplatin concentrations in the kidneys in the presence and absence of pevonedistat to investigate the possibility that pevonedistat could directly impact renal levels of cisplatin. We focused our study on defining pharmacodynamic mechanisms and demonstrated that pevonedistat likely attenuates cisplatin-mediated nephrotoxicity by alleviating oxidative stress. This is further supported by the lack of change in expression of the cisplatin transporters OCT2 and CTR1 following pevonedistat treatment. A future study will directly measure the effects of cisplatin concentrations in the kidney as well as on the activity of key cisplatin transporters to determine whether other mechanisms may alter cisplatin-mediated renal toxicity. Cisplatin frequently induces kidney injury through tubular necrosis (21). It is interesting that we also observed glomerular collapse in our study. It is possible that persistent oxidative stress due to repeated doses of cisplatin contributed to this effect. Further investigation of this phenomenon is warranted.

Pevonedistat is currently being clinically developed as an anticancer agent. Promising clinical activity has been observed in the treatment of patients with myelodysplastic syndromes and acute myeloid leukemia (46, 47). We and others have also shown that pevonedistat has significant activity against a variety of solid tumors including HNSCC (15, 48–50). However, our current study establishes a potential novel application for pevonedistat to protect kidney tissue from cisplatin-induced toxicity. Given the prevalent use of cisplatin in a wide range of chemotherapy regimens, this approach could have a transformative impact for the management of multiple cancers if clinically validated. Targeted inhibition of NEDDylation as a precision strategy to protect normal cells from injury could also have much broader applications beyond nephrotoxicity. TXNIP is highly expressed in myocardial and intestinal epithelial cells, both of which are susceptible to off-target toxicities of a variety of chemotherapeutic agents (51–53). Therefore, pevonedistat may have additional utility as a chemoprotective agent.

Our work demonstrated that targeted inhibition of NEDDylation with pevonedistat protects normal kidney cells from oxidative stress–induced toxicity following cisplatin therapy while simultaneously enhancing its antitumor activity. We also determined that differential regulation of TXNIP in normal and cancer cells may provide an opportunity for precision therapeutic targeting to improve efficacy and diminish potential toxicity. A clinical trial further investigating the ability of pevonedistat to protect against cisplatin-induced nephrotoxicity is being planned.

## Supplementary Material

Supplementary Figure S1 qRT-PCR of selected genes in the oxidoreductase pathway.

Supplementary Figure S2 Pevonedistat stabilizes NRF2 expression in RPTECs.

Supplementary Figure S3 Knockdown of NRF2 blunts pevonedistat-mediated protection from cisplatin.

Supplementary Figure S4 Pevonedistat does not alter OCT2 or CTR1 expression.

Supplementary Figure S5 Quantification of IHC staining.

Supplementary Table TS1 Genes altered by 2 fold change or more by pevonedistat treatment.

Supplementary Table TS2 Significantly upregulated oxidative stress response genes by pevonedistat.

Supplementary Table TS3 Combination indices following treatment with pevonedistat and cisplatin.

Supplementary Table TS4 Median survival time of FaDu tumor bearing mice.
