# Supplementary material for: Targeting NEDDylation is a Novel Strategy to Attenuate Cisplatin-induced Nephrotoxicity
Source: Cancer Res Commun. 2023 Feb 13;3(2):245–57. doi: 10.1158/2767-9764.CRC-22-0340 (PMC9973416; doi:10.1158/2767-9764.CRC-22-0340)
Supplement: Supplementary Figure S1 — qRT-PCR of selected genes in the oxidoreductase pathway. [file crc-22-0340-s01.pdf]

Supplementary Figure S1

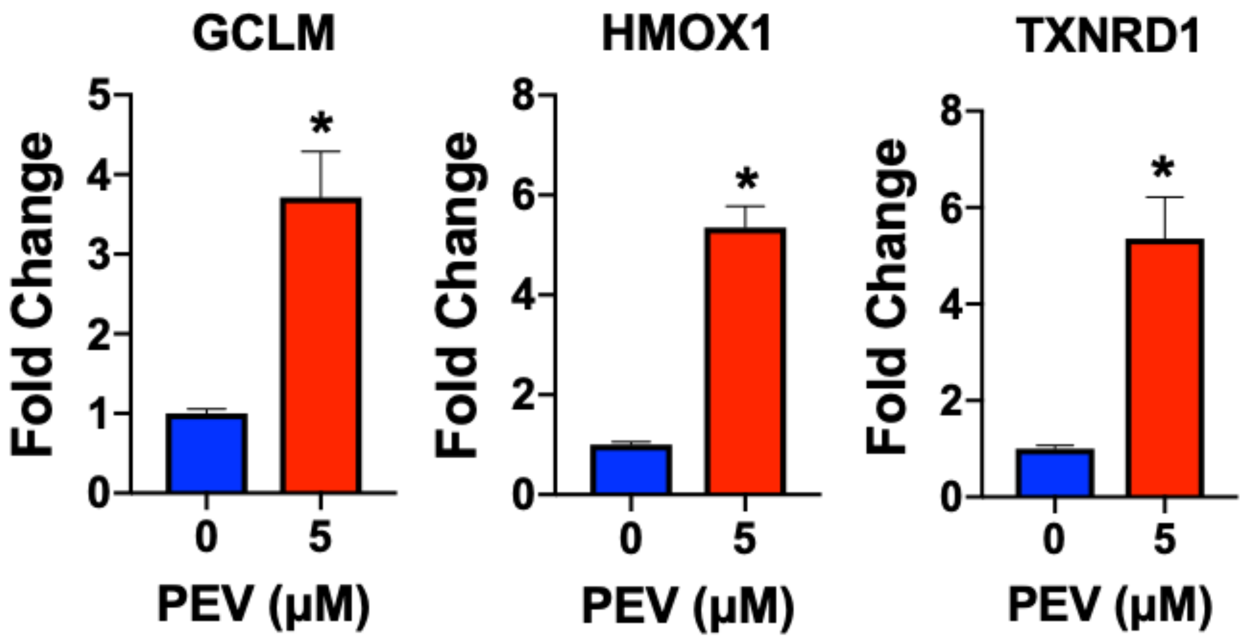

Supplementary Figure S1. Quantitative RT-PCR (qRT-PCR) confirms enrichment of genes in the stress response and oxidoreductase pathways. RNA was collected from control and pevonedistat treated RPTEC cells and *GCLM*, *HMOX1*, and *TXNRD1* were measured by qRT-PCR. Results demonstrate that these genes are significantly upregulated compared to controls. Mean ± SD, n = 3. * $p < 0.05$.
