# Supplementary material for: Targeting NEDDylation is a Novel Strategy to Attenuate Cisplatin-induced Nephrotoxicity
Source: Cancer Res Commun. 2023 Feb 13;3(2):245–57. doi: 10.1158/2767-9764.CRC-22-0340 (PMC9973416; doi:10.1158/2767-9764.CRC-22-0340)
Supplement: Supplementary Figure S2 — Pevonedistat stabilizes NRF2 expression in RPTECs. [file crc-22-0340-s02.pdf]

Supplementary Figure S2

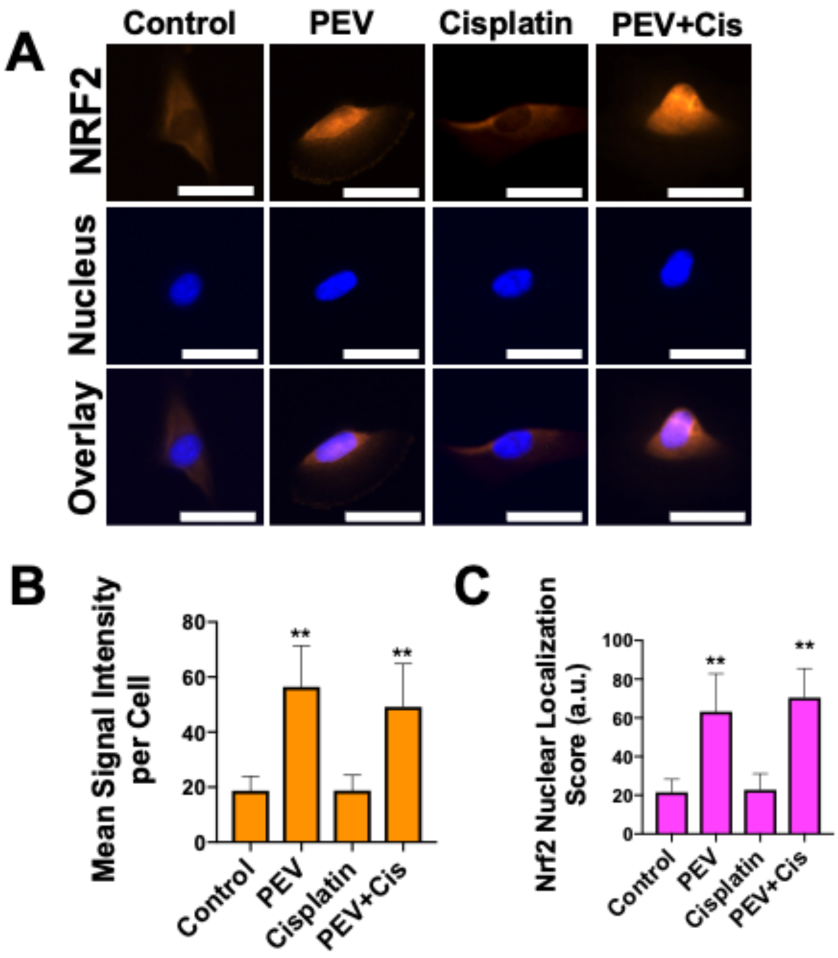

Supplementary Figure S2. Pevonedistat stabilizes NRF2 expression in RPTECs. **(A)** RPTECs were treated with 300 nM PEV, 15 μM cisplatin, or the combination for 24 hours. Representative images are displayed. **(B)** Mean NRF2 signal intensity was measured via ImageJ. Mean ± SD, n = 17 cells per condition. **Indicates significant difference from control; $p < 0.01$. **(C)** Mean NRF2 nuclear signal intensity co-localized with DAPI counterstain was measured using ImageJ. Mean ± SD, n = 17 cells per condition. **Indicates significant difference from control; $p < 0.01$.
