# Supplementary material for: Targeting NEDDylation is a Novel Strategy to Attenuate Cisplatin-induced Nephrotoxicity
Source: Cancer Res Commun. 2023 Feb 13;3(2):245–57. doi: 10.1158/2767-9764.CRC-22-0340 (PMC9973416; doi:10.1158/2767-9764.CRC-22-0340)
Supplement: Supplementary Figure S3 — Knockdown of NRF2 blunts pevonedistat-mediated protection from cisplatin. [file crc-22-0340-s03.pdf]

Supplementary Figure S3

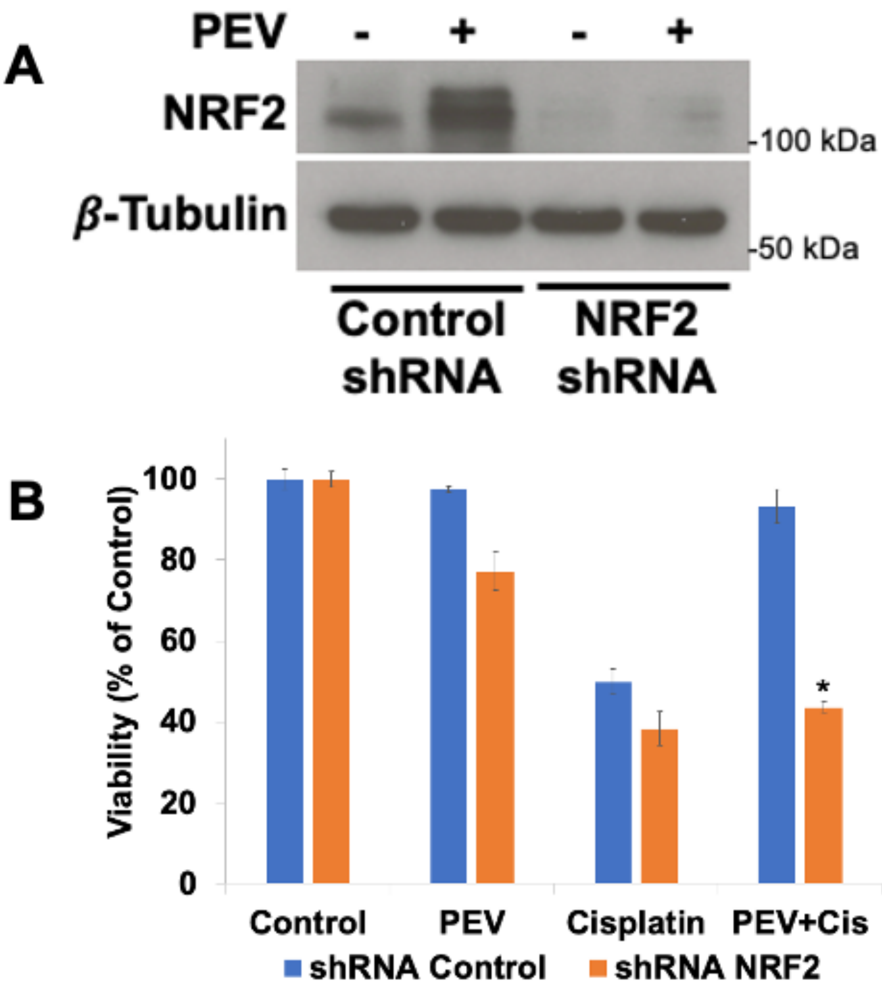

Supplementary Figure S3. Knockdown of NRF2 significantly blunts pevonedistat-mediated protection from cisplatin toxicity. **(A)** RPTEC cells were infected with lentiviral control or NRF2 shRNA. Knockdown of NRF2 at baseline and following pevonedistat treatment was confirmed by immunoblotting. **(B)** Control and NRF2 shRNA cells were treated with 100 nM pevonedistat, 600 nM cisplatin, or the combination for 72 hours. Cell viability was measured by MTT assay. Mean ± SD, n = 3, $p < 0.05$. *Indicates significant difference from control shRNA cells treated with the combination of pevonedistat and cisplatin.
