# Supplementary material for: Targeting NEDDylation is a Novel Strategy to Attenuate Cisplatin-induced Nephrotoxicity
Source: Cancer Res Commun. 2023 Feb 13;3(2):245–57. doi: 10.1158/2767-9764.CRC-22-0340 (PMC9973416; doi:10.1158/2767-9764.CRC-22-0340)
Supplement: Supplementary Figure S4 — Pevonedistat does not alter OCT2 or CTR1 expression. [file crc-22-0340-s04.pdf]

Supplementary Figure S4

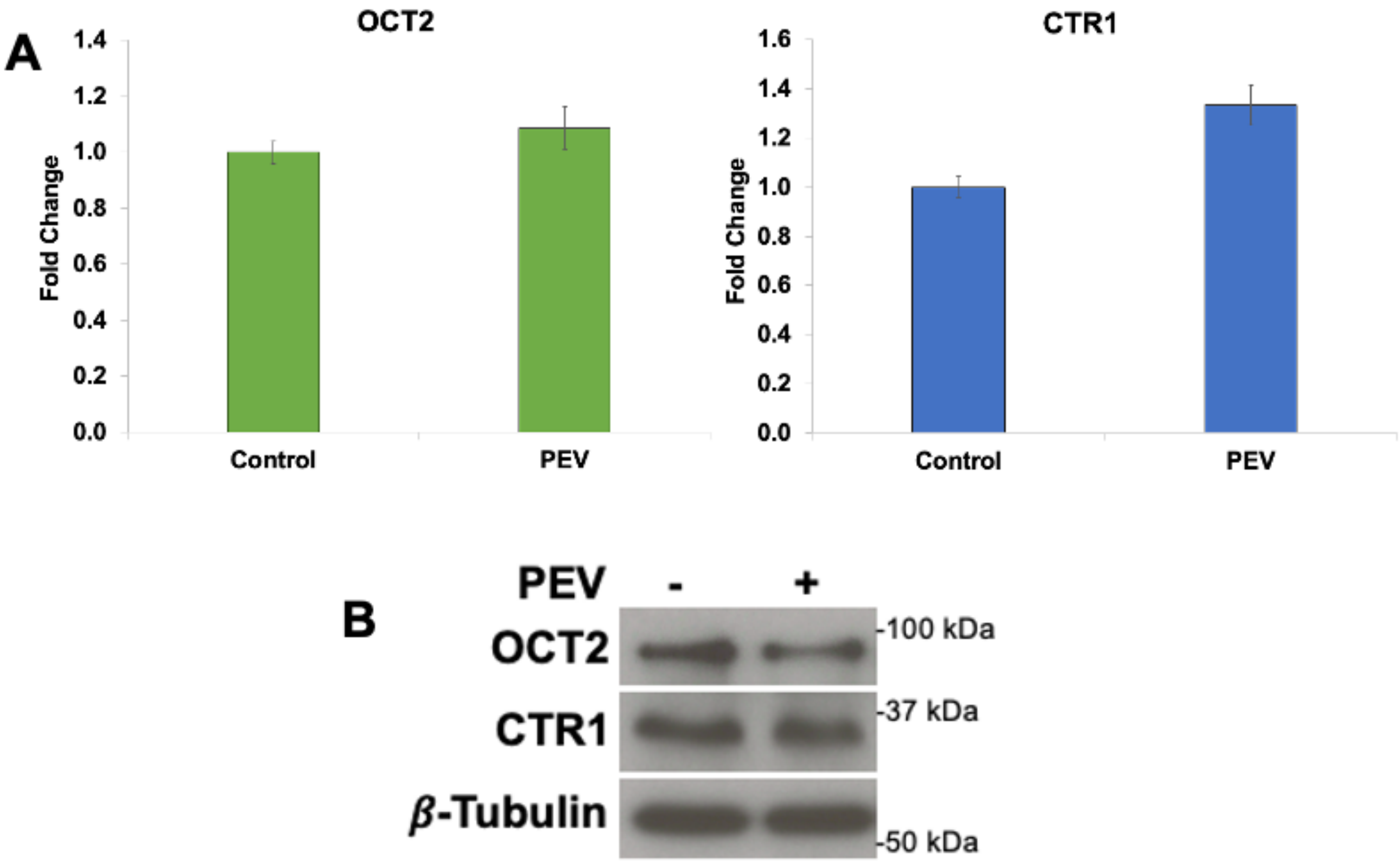

Supplementary Figure S4. Pevonedistat does not significantly alter OCT2 or CTR1 levels in RPTEC cells. **(A)** RPTECs were treated with 5 μM pevonedistat for 48 hours. OCT2 and CTR1 expression was measured by qRT-PCR. **(B)** RPTEC cells were treated with 5 μM pevonedistat for 48 hours and OCT2 and CTR1 protein levels were detected by immunoblotting.
