# Supplementary material for: Targeting NEDDylation is a Novel Strategy to Attenuate Cisplatin-induced Nephrotoxicity
Source: Cancer Res Commun. 2023 Feb 13;3(2):245–57. doi: 10.1158/2767-9764.CRC-22-0340 (PMC9973416; doi:10.1158/2767-9764.CRC-22-0340)
Supplement: Supplementary Figure S5 — Quantification of IHC staining. [file crc-22-0340-s05.pdf]

Supplementary Figure S5

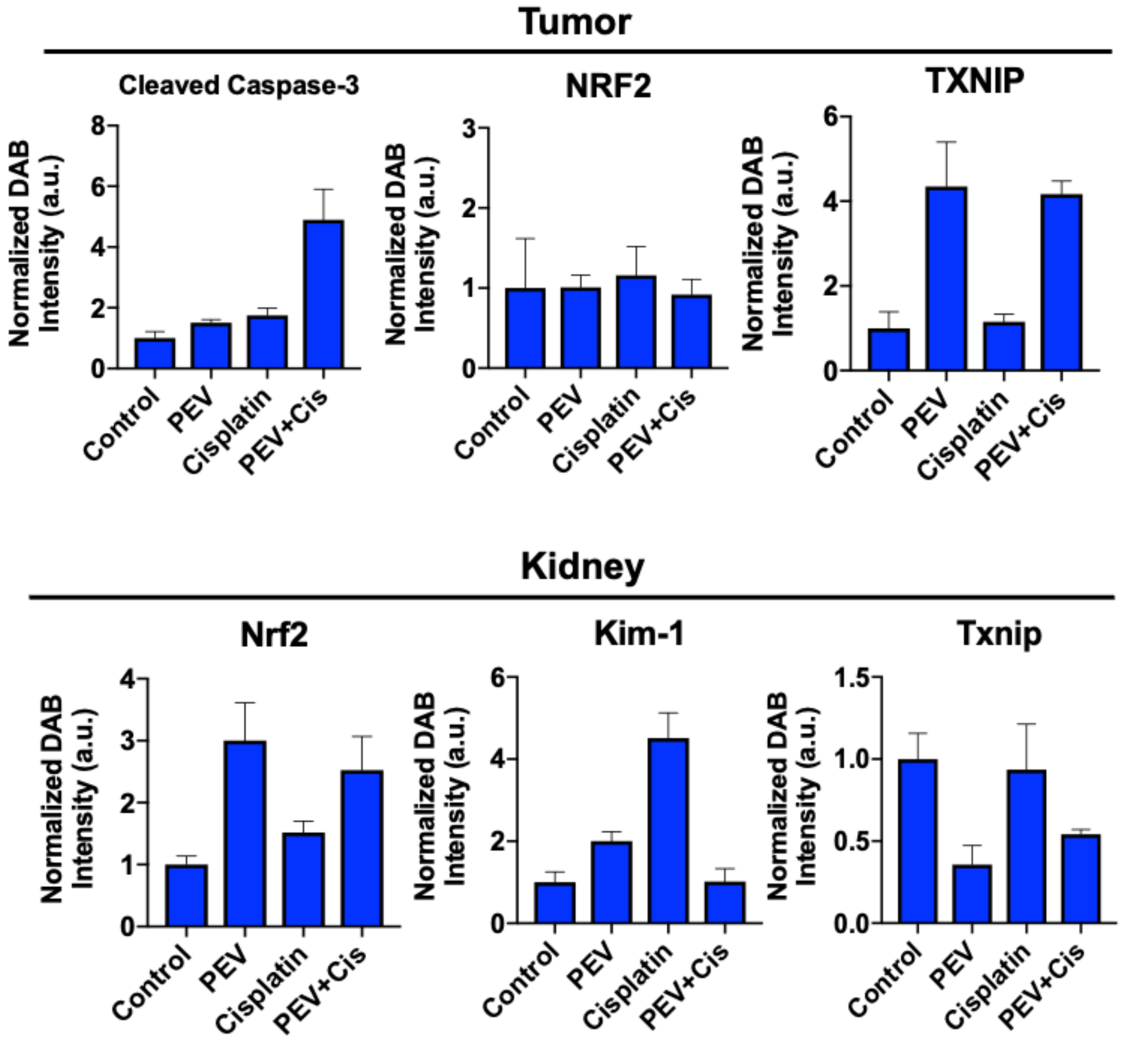

Supplementary Figure S5. Quantification of IHC staining intensities were quantified using ImageJ color deconvolution software. Mean ± SD, n = 5 images per condition.
